# Chikungunya Virus, Southeastern France

**DOI:** 10.3201/eid1705.101873

**Published:** 2011-05

**Authors:** Marc Grandadam, Valérie Caro, Sébastien Plumet, Jean-Michel Thiberge, Yvan Souarès, Anna-Bella Failloux, Hugues J. Tolou, Michel Budelot, Didier Cosserat, Isabelle Leparc-Goffart, Philippe Desprès

**Affiliations:** Author affiliations: Institut Pasteur, Paris, France (M. Grandadam, V. Caro, J.-M. Thiberge, A.-B. Failloux, P. Desprès);; Institut de Recherche Biomédicale des Armées, Marseille, France (S. Plumet, H.J. Tolou, I. Leparc-Goffart);; Institut de Veille Sanitaire, Saint-Maurice, France (Y. Souarès);; Private Consulting Rooms, Saint-Raphaël, France (M. Budelot, D. Cosserat)

**Keywords:** Chikungunya, vector-borne infections, viruses, emergence, autochthonous transmission, southeastern France, dispatch

## Abstract

In September 2010, autochthonous transmission of chikungunya virus was recorded in southeastern France, where the *Aedes albopictus* mosquito vector is present. Sequence analysis of the viral genomes of imported and autochthonous isolates indicated new features for the potential emergence and spread of the virus in Europe.

Chikungunya virus (CHIKV; *Togaviridae*, genus *Alphavirus*), transmitted to humans by the bite of *Aedes* spp*.* mosquito, leads to an acute fever associated with an arthromyalgic syndrome (*1*). CHIKV outbreaks occurred after the virus’s recent expansion in Africa, the Indian Ocean, India, and Southeast Asia. Phylogenetic analyses have demonstrated 3 distinct lineages of CHIKV strains: West Africa, Asia, and East/Central/South Africa (ECSA) (*2**,**3*). Strains from the Indian Ocean and India segregate into 2 independent sublineages that presumably derive from an East African ancestral genotype (*2**,**3*). Until recently, the *Ae. aegypti* mosquito was widely accepted as the main urban vector of CHIKV. However, the *Ae*. *albopictus* mosquito was extensively implicated in CHIKV transmission during the 2005–06 outbreak in Réunion Island (*1*).

## The Study

Reinforced surveillance systems aimed at monitoring the introduction of CHIKV have been implemented in 6 departments in southeastern France, including the Var department, where *Ae. albopictus* has spread since its introduction in 2004, presumably from northern Italy (*4*). On August 29, 2010, a 7-year-old girl (patient 1) with acute febrile syndrome, headache, and abdominal pain sought treatment in the city of Fréjus (Var) 1 day after she had returned from Rajasthan, India. Continuous CHIKV circulation in northern India districts has been reported during 2009–2010 (www.promedmail.org). The patient’s serum sample was found positive for CHIKV infection by reverse transcription–PCR (RT-PCR) (*5**,**6*). Three weeks after the notification of patient 1, another young girl (patient 2) experienced clinical symptoms that began on September 18 with fever, arthralgia, backache, headache, and retro-orbital pain. Patient 2 had no history of travel in areas endemic for CHIKV. She resided 2.5 km from patient 1. The serum specimen was positive for CHIKV diagnosis. Patient 2’s physician reported that a young girl (patient 3), a close friend of her patient, showed clinical symptoms compatible with CHIKV infection at the same time. Patient 3, who lives near patient 1, had invited patient 2 to spend the night of September 15 at her home. The 2 children reported numerous mosquito bites. A serum sample from patient 3 was collected 1 week after onset of fever and monoclonal antibody capture ELISA detected high titers of specific anti-CHIKV immunoglobulin M. The serum sample also showed a weak RT-PCR signal for CHIKV. Given that patients 2 and 3 did not report any recent travel to areas endemic for CHIKV, their illnesses were classified as autochthonous cases of CHIKV infection. No complications were recorded, but all 3 patients had persistent weakness and joint pain 3 months after the acute phase.

High densities of *Ae. albopictus* mosquitoes have been found in the Var department since 2008. Intensive mosquito control measures, including spraying for adult mosquitoes and destroying breeding sites, were undertaken around the patients’ residences and areas visited by confirmed case-patients. No further cases were found by the active case finding system (a local physician and laboratories network) implemented for 45 days after the declaration of the last autochthonous case.

A molecular study of France/2010 CHIKV strains isolated in Fréjus obtained from patients 1 (imported case) and 2 (autochthonous case) was performed. Viral genomic RNA was extracted from CHIKV grown once in mosquito C6/36 cells and then subjected to RT-PCR amplification by using a set of primers targeting the structural genes of CHIKV (*7*). Paired sequence analysis of the E2–6K–E1 junction showed that the 2 France/2010 CHIKV strains display a divergence rate <0.05% at the nucleotide level, whereas 100% identity was observed at the amino acid level. Phylogenetic analysis demonstrated that these viral strains belong to a cluster that is closely related to strains from India within the ECSA lineage (Figure). The France/2010 CHIKV isolate from patient 2 might be derived from an Indian strain introduced by patient 1 (index case). Genotypes E2-211T, E2-312M, E2-386A, 6K-8I, and E1-284E that are found in the currently circulating strains belonging to the ECSA lineage were identified in France/2010 CHIKV isolates (*2**,**3**,**7*). These isolates also display the genotype E1-211E specifically shared by viral strains belonging to the Asian phylogenetic group (Table). The residue Ala at position E2-264 has not been previously described in any CHIKV strains.

**Figure:**
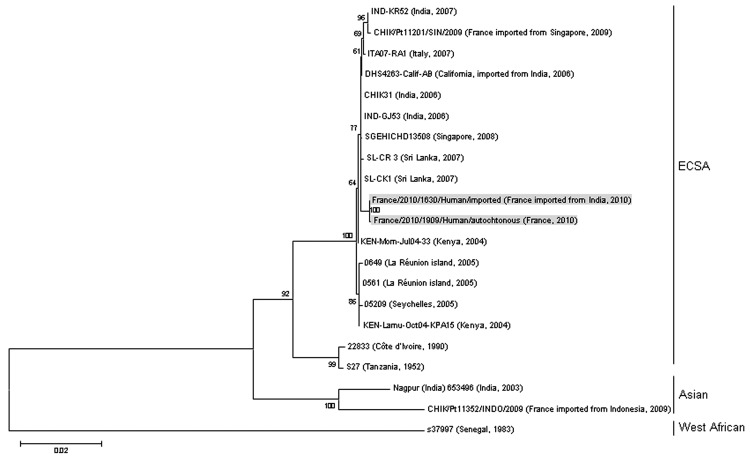
Phylogenetic relationships among chikungunya virus isolates from cases of chikungunya fever in France, based on complete E2-6K-E1 nucleotide sequence (2,771 nt) analysis. Gray shading indicates imported and autochthonous strains. Sequence alignments were performed by using BioNumerics version 5.1 (www.applied-maths.com). Phylogenetic analysis was inferred by using the maximum-likelihood method as implemented in MEGA version 5 software (www.megasoftware.net). Bootstrap support values (1,000 replicates) are indicated at major nodes. The sequence of the strains from France described in this study has been deposited in GenBank (accession number pending); other sequences were retrieved from GenBank. Scale bar indicates number of base substitutions per site. ECSA, east/central/south Asia.

**Table:** Relevant amino acid substitutions identified between France/2010 CHIKV isolates (autochthonous and imported cases) versus a selection of CHIKV strains*

Strain	Country	Year	Protein position							
E2-60	E2-162	E2-211	E2-264†	E1-211	E1-226	E1-269	E1-284
France/2010/1630/human/ imported (a)	France (India)	2010	D	A	T	**A**	**E**	**A**	V	E
France/2010/1909/human/ autochthonous (a)	France	2010	D	A	T	**A**	**E**	**A**	V	E
22833 (a)	Côte d'Ivoire	1990	D	A	I	V	K	A	M	D
CHIK31 (a)	India	2006	D	A	T	V	K	A	V	E
05209 (a)	Seychelles	2005	D	A	T	V	K	A	V	E
0561 (a)	Réunion	2005	D	A	T	V	K	A	V	E
0649 (a)	Réunion	2005	D	A	T	V	K	V	V	E
DHS4263-Calif-AB (a)	California (India)	2006	D	A	T	V	K	A	V	E
IND-GJ53 (a)	India	2006	D	A	T	V	K	A	V	E
IND-KR52 (a)	India	2007	D	A	T	V	K	V	V	E
ITA07-RA1 (a)	Italy	2007	D	A	T	V	K	V	V	E
KEN-Lamu-Oct04-KPA15 (a)	Kenya	2004	D	A	T	V	K	A	V	E
KEN-Mom-Jul04-33 (a)	Kenya	2004	D	A	T	V	K	A	V	E
S27 (a)	Tanzania	1952	D	A	I	V	K	A	M	D
SGEHICHD13508 (a)	Singapore	2008	D	A	T	V	K	A	V	E
CHIK/Pt11201/SIN/2009 (a)	France (Singapore)	2009	D	A	T	V	K	V	V	E
SL-CK1 (a)	Sri Lanka	2007	D	A	T	V	K	A	V	E
SL-CR 3 (a)	Sri Lanka	2007	D	A	T	V	K	A	V	E
Nagpur (India) 653496 (b)	India	2003	D	A	T	V	E	A	M	D
CHIK/Pt11352/INDO/2009 (b)	France (Indonesia)	2009	D	A	T	V	E	A	M	D
s37997 (c)	Senegal	1983	D	A	T	V	K	A	V	D

*Molecular signatures were based on the analysis of complete amino acid sequence E2-6K-E1 (923 aa). The numbering of amino acid positions refers to the African isolate S27 (GenBank access no. AF369024). Residues in **boldface** indicate critical aa changes. Letters in parentheses after strain names refer to East/Central/South Africa (a), Asia (b) and West Africa (c) phylogroups. Country names in parentheses identify source of imported case. CHIKV, chikungunya virus. †The amino acid substitution was unique to France/2010 CHIKV isolates.

Recent attention has focused on the predominant role of E1 and E2 proteins in successful CHIKV infection of the anthropophilic *Ae. albopictus* (*2**,**3**,**7**,**8*). Vector competence experiments with La Réunion/2006 CHIKV isolates demonstrated the importance of the newly acquired E1-Ala226Val substitution for efficient transmission by *Ae. albopictus* mosquitoes during the 2006 outbreak in Réunion Island (*7**–**10*). Italy/2007 CHIKV strains also exhibited the signature E1-226V genotype (*11*). *Ae.* *albopictus* from northern Italy and from southeastern France showed disseminated infection rates ranging from 75%–90% for CHIKV strains with E1-226V (*10*). The 2 France/2010 CHIKV strains isolated in Fréjus have Ala at position E1-226 (Table). The presence of an Asp residue at position E2-60, found in most of the ECSA CHIKV strains, may in part counterbalance the less favorable transmission of E1-226A strain in *Ae. albopictus* (Table). The Thr residue at position E2-211 potentiates the infectivity of CHIKV in *Ae. albopictus* mosquitoes only in synergy with E1-226V. The presence of E2-211T in CHIKV isolates from France underlines the risk for emergence of a fully adapted viral variant if the E1-226V genotype was selected during continuous transmission within *Ae. albopictus* populations in France (*7**,**8**,**9*).

## Conclusions

The efficient CHIKV transmission in Italy and southeastern France sheds new light on its dissemination potential in Europe from 1 index case, regardless of the viral genetic background and mosquito species in the region of origin of the imported CHIKV (*1**,**10**,**11*). In emerging regions, such as Italy and Réunion Island, where the seroprevalence in the population was <50%, no confirmed cases were recorded for years after an outbreak. Italy has not reported any new autochthonous cases since 2007. However, >2 years passed since the end of the epidemic in Réunion Island before a local transmission of CHIKV was again detected. In Europe where sylvatic cycles are absent, vertical transmission may participate in the maintenance and/or cyclic reemergences of CHIKV. This critical issue remains to be investigated in diapausing temperate populations of *Ae. albopictus* that may have more efficient vertical transmission than mosquito populations in eastern Italy and tropical regions (*11**,**12*).

In 2010, southeastern France faced the concomitant emergences of dengue virus (DENV) and CHIKV (*13*). For each of these viruses, only 2 autochthonous infections were confirmed, which suggests that rapid detection and control measures implemented around imported and autochthonous cases have been efficient. A recent report mentioned the dual emergence of CHIKV and DENV in southeastern France and urged the implementation of specific surveillance and response measures to reduce the risk for arbovirus emergence (*14*). Since 2006, a specific chikungunya/dengue national preparation and response plan based on rapid detection and investigation of imported and suspected autochthonous cases, mosquito control measures, and efficiency evaluation in the treated areas has been activated from May through November and then modified after annual debriefing meetings involving all partners. In 2010, this model proved to be well adapted to the early detection and control of CHIKV and DENV. Considering the expanding global distribution of *Ae. albopictus* mosquitoes and the successful emergence of CHIKV in Italy and France, reinforced surveillance and response to CHIKV and DENV dissemination should become a higher priority in Europe (*15*).
